# Sensor Fusion and State Estimation of IoT Enabled Wind Energy Conversion System

**DOI:** 10.3390/s19071566

**Published:** 2019-04-01

**Authors:** Md. Noor-A-Rahim, M. O. Khyam, Xinde Li, Dirk Pesch

**Affiliations:** 1 School of Computer Science and IT, University College Cork, T12 K8AF Cork, Ireland; d.pesch@cs.ucc.ie; 2 Nimbus Centre, Cork Institute of Technology, T12 P928 Cork, Ireland; 3 Department of Mechanical Engineering, Virginia Tech, Blacksburg, VA 24061, USA; mok@vt.edu; 4 School of Automation, Southeast University, Nanjing 210096, China; xindeli@seu.edu.cn

**Keywords:** Wind energy, Sensor fusion, State estimation, Internet of Things (IoT), Renewable energy

## Abstract

The use of renewable energy has increased dramatically over the past couple of decades. Wind farms, consisting of wind turbines, play a vital role in the generation of renewable energy. For monitoring and maintenance purposes, a wind turbine has a variety of sensors to measure the state of the turbine. Sensor measurements are transmitted to a control center, which is located away from the wind farm, for monitoring and maintenance purposes. It is therefore desirable to ensure reliable wireless communication between the wind turbines and the control center while integrating the observations from different sensors. In this paper, we propose an IoT based communication framework for the purpose of reliable communication between wind turbines and control center. The communication framework is based on repeat-accumulate coded communication to enhance reliability. A fusion algorithm is proposed to exploit the observations from multiple sensors while taking into consideration the unpredictable nature of the wireless channel. The numerical results show that the proposed scheme can closely predict the state of a wind turbine. We also show that the proposed scheme significantly outperforms traditional estimation schemes.

## 1. Introduction

Demand for renewable energy is rapidly increasing in order to counteract progress of global warming and diminishing natural resources. Wind turbines play a significant role in renewable energy generation. It is predicted that wind farms, consisting of many wind turbines, will supply about 10% of total electricity by the year 2020 [1,2]. Usually, wind turbines are placed in remote areas where plenty of wind is available. Examples of such places are off or near the seashore or on mountains. For maintenance and monitoring purposes, various sensors are embedded into a wind turbine to measure the state of the turbine. However, convenient monitoring of the state requires remote communication connectivity to the wind turbines. A viable solution to this problem can be an Internet of Things (IoT) based network [3,4,5]. Integration of the wind turbines into an IoT network can enable a real-time monitoring system to monitor turbine performance and detect faults so that technicians can take necessary actions promptly. As communication links between the wind turbines and the Internet will likely take place over wireless communication channels, a reliable communication framework is required between the wind turbines and wireless base stations or access points connected to the Internet. On top of that, to process and combine the multiple sensor readings, a fusion algorithm needs to be developed for accurate state estimation while taking into account the uncertainty of the wireless channel.

In previous works, various algorithms and models have been developed to estimate the state of the wind turbine and examples of such works are [6,7,8,9,10,11,12,13,14,15,16] (see Table 1). A sensor selection procedure for estimation of wind turbine rotor state and wind-induced load state was presented in [6]. Considering nonlinear observers, a wind turbine dynamic state and parameter estimation technique was described in [8]. In [9], a semi-empirical method for wind turbine state estimation was proposed. In conjunction with the Newton-Raphson method, a Kalman filter based estimation technique was described in [10] to estimate the aerodynamic torque acting on the rotor of the wind turbine. To overcome drawbacks due to the decoupling effects, an extended state estimation method was proposed in [11], where the models for the different wind turbine technologies were incorporated. A type of non-linear state estimator called particle filter was utilized in [12] to estimate the wind turbine parameters: tower top displacement, tower stop velocity and the rotor speed. More recently, authors in [13,14,15,16] studied dynamic state estimation of doubly fed induction generator (DFIG) based wind turbine and permanent magnet synchronous generator (PMSG) based wind turbine. However, most of the previous work did not take into account the impact of an unreliable wireless channel (between the wind turbines and the remote controller) on the state estimation of the wind turbine. Moreover, IoT based network can play a vital role in enabling a reliable and real-time state estimation and controlling of wind turbines. Although the potentials of IoT were discussed in a few studies [3,4,5,17], not much is known regarding wireless communication framework of IoT based state estimation of wind turbine. On the other hand, the embedded sensors in a wind turbine measure different components of the turbine independently. Thus, an appropriate sensor fusion algorithm is required to combine the readings of different sensors for accurate state estimation. Note that previous research, including [18,19,20,21,22,23,24] considered sensor fusion techniques for generic observable plants. However, to the best of our knowledge, no work has considered sensor fusion techniques in the context of turbine-based wind energy systems.

To address these shortcomings, this paper studies sensor fusion and state estimation of wind turbines over a wireless IoT network. We present a communication framework for wind turbines and remote IoT components. To achieve a reliable communication between the wind turbines and the remote observer, we present a repeat-accumulate coded transmission scheme over a wireless channel. To exploit different observations from the sensors, we propose a Kalman filter based sensor fusion technique while taking into consideration the error events caused by the wireless channel. Through the numerical results, we show that the proposed estimation technique can closely predict the state of the wind turbine. We also show that the proposed scheme significantly outperforms traditional state estimation techniques.

The remainder of this paper is organized as follows. In Section 2, we present an IoT enabled wind energy conversion system and linearized state space model of wind turbine’s induction motor. The proposed communication framework is presented in Section 3. In Section 4, we propose Kalman filter based sensor fusion technique. We present the simulation results of the proposed fusion scheme in Section 5 along with the improvement over the traditional scheme.

## 2. IoT Enabled Wind Energy Conversion System and State Space Model

**Figure 1:**
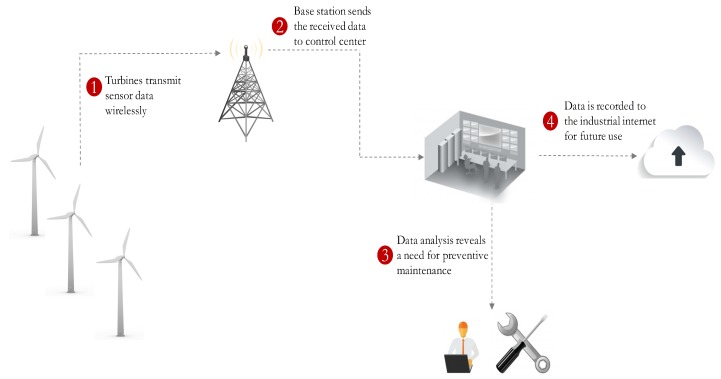
Use of Internet of Things (IoT) network for transmitting data from turbines to control center and technicians.

In general, wind farms are situated in remote locations and thus the control centers are typically located several hours away from the wind farms. Remote data communication connectivity through the Internet in the form of an IoT network can help the control centers to monitor the state of a wind turbine and to control a wind turbine’s operation. Due to the remote location of wind turbines, the IoT network connectivity requires wireless networks such as cellular or satellite networks. An integrated scenario comprised of wind turbines and a wireless IoT network [25] is depicted in Figure 1.

A wind turbine system consists of induction generator, rotors, gearbox, and matrix converter [26,27]. The matrix converter connects the induction generator with the power grid and controls the output power delivered to the grid. In this paper, we track the output current components of an induction generator in a wind turbine. We adopt the state space model reported in [28], where a fixed-speed wind turbine is considered. Authors in [28] utilized the current model to characterize the induction generator and the following assumptions were considered for the sake of simplicity: (1) the stator current is assumed to be negative when it flows toward the machine; (2) a synchronous reference frame was considered to derive the equations; (3) the d-axis is 90 deg behind the q-axis (Direct axis (d-axis) is the axis of the stator/rotor’s salient pole and quadrature axis (q-axis) is the axis in quadrature or perpendicular to the stator/rotor’s salient pole.). With the above assumptions, we adopt the linearized state space current model of induction generator, which is described by the following equation:

(1) $$\begin{array}{c} \dot{X}=AX+BU+\Gamma {𝒩}_{p} \end{array}$$

where $X={\left[i_{ds}\;i_{qs}\;i_{dr}\;i_{qr}\right]}^{T}$ is the state of the induction generator, $U={\left[v_{ds}\;v_{qs}\;v_{dr}\;v_{qr}\right]}^{T}$ is control input, Γ is a constant matrix, and ${𝒩}_{p}$ is process noise. We model the process noise as Gaussian noise with zero-mean and covariance matrix Q. In X, $i_{ds}$ and $i_{qs}$ are the stator currents in the *d* and *q* axes, respectively; $i_{dr}$ and $i_{qr}$ are the rotor currents in the *d* and *q* axes, respectively. In U, $v_{ds}$ and $v_{qs}$ are the stator voltages in the *d* and *q* axes, respectively; $v_{dr}$ and $v_{qr}$ are the stator voltages in the *d* and *q* axes, respectively. With the specifications of a single-cage induction generator, A and B are characterized by [28,29],

$$A=\frac{\omega_{b}}{X_{s}X_{r}\gamma }\left[\begin{array}{cccc} -R_{s}X_{r} & \alpha_{1}\omega_{s} & -R_{r}X_{m} & -\beta_{r}\omega_{s}\\ -\alpha_{1}\omega_{s} & -R_{s}X_{r} & \beta_{r}\omega_{s} & -R_{r}X_{m}\\ -R_{s}X_{m} & \beta_{s}\omega_{s} & -R_{r}X_{s} & -\alpha_{2}\omega_{s}\\ -\beta_{s}\omega_{s} & -R_{s}X_{m} & \alpha_{2}\omega_{s} & -R_{r}X_{s} \end{array}\right]$$

$$B=\frac{\omega_{b}}{X_{s}X_{r}\gamma }\left[\begin{array}{cccc} -X_{r} & 0 & X_{m} & 0\\ 0 & -X_{r} & 0 & X_{m}\\ -X_{m} & 0 & X_{s} & 0\\ 0 & -X_{m} & 0 & X_{s} \end{array}\right]$$

where $\omega_{b}$, $\omega_{s}$, $\omega_{r}$ are base, stator, rotor angular speeds, respectively; *s* is the slip defined by $s=\frac{\omega_{s}-\omega_{r}}{\omega_{s}}$; $R_{s}$, $R_{r}$ Stator, rotor resistances, respectively; $X_{s}$, $X_{r}$, $X_{m}$ stator, rotor, magnetizing reactances, respectively; $\alpha_{1}=X_{s}X_{r}-sX_{m}^{2}$, $\alpha_{2}=X_{m}^{2}-sX_{s}X_{r}$, $\beta_{s}=X_{m}X_{s}\left(1-s\right)$, $\beta_{r}=X_{m}X_{r}\left(1-s\right)$, $\gamma =1-\frac{X_{m}^{2}}{X_{r}X_{s}}$.

For the sake of simplicity, we discretise Equation (1) in the following form [30]:

(2) $$\begin{array}{cc} X(t+1) & =A_{d}X\left(t\right)+B_{d}U\left(t\right)+\Gamma {𝒩}_{p} \end{array}$$

where $A_{d}$ and $B_{d}$ are obtained by,

$$\begin{array}{c} A_{d}=\exp\left(A\Delta t\right)≃\left(I\right)+A\Delta t\\ B_{d}=\int_{0}^{\Delta t}\exp\left(Az\right)Bdz≃B\Delta t \end{array}$$

where Δt is the step size used for discretization.

## 3. Proposed Communication Framework

**Figure 2:**
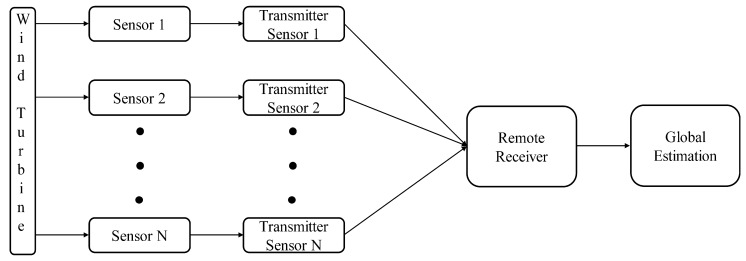
Communication framework of multi-sensor wind turbine.

In a wind turbine, different types of sensors are embedded to measure the components of the wind turbine, for example, generated current, voltage, rotor speed, etc. Let ${𝒪}_{i}\left(t\right)$, i=1,2,…,N, be the measured state by the $i^{th}$ sensor of the wind turbine. We define ${𝒪}_{i}\left(t\right)$ by,

$$\begin{array}{c} {𝒪}_{i}\left(t\right)=C_{i}X\left(t\right)+{𝒩}_{m_{i}}, \end{array}$$

where $C_{i}$ is the measurement/sensing matrix of sensor *i* and ${𝒩}_{m_{i}}$ is the observed noise during the measurement at sensor *i*. Similar to the process noise, we model the measurement noise as Gaussian noise with zero-mean and co-variance $R_{i}$. The measured state is sent periodically to the control center for the appropriate action to be taken. Due to the remote placement of wind turbines there often is no direct communication link between a wind turbine and the control center. In most cases, a transmitter in the wind turbine communicates with a nearby base station, from where the message is relayed to the control center. We assume that the communication link between the base station and the control center is reliable as it is part of a fixed backbone network. However, due to the wireless communication channel between the wind turbine and the base station it is challenging to maintain reliable data communication, although reliable communication is highly desirable for accurate state estimation and control applications. To achieve reliable communication between the wind turbine and the base station we propose the following communication strategy. We define the observed state as ${𝒪}_{i}\left(t\right)=\left[o_{i1},o_{i2},\ldots ,o_{ip}\right]$, where $o_{ij}\left(t\right)$ is the $j^{th}$ component of X, which is measured by the $i^{th}$ sensor. Each component of ${𝒪}_{i}\left(t\right)$ is mapped and quantized into *K* bits. The bit block corresponding to component *j* is represented by $b_{ij}\left(t\right)\in {\left\{0,1\right\}}^{K}$. A repeat-accumulate code is then applied over $b_{ij}\left(t\right)$ to generate a code word $c_{ij}\left(t\right)\in {\left\{0,1\right\}}^{\frac{K}{\eta }}$, where η is the rate of the code. All code words are arranged serially to form $m_{i}\left(t\right)=\left[c_{i1}\left(t\right),c_{i2}\left(t\right),\ldots ,c_{ip}\left(t\right)\right]$. After modulating the $m_{i}\left(t\right)$ onto the wireless carrier signal, the resultant carrier signal $s_{i}\left(t\right)$ is then transmitted from the wind turbine to the base station. Let ${\hat{s}}_{i}\left(t\right)$ be the received signal at the base station, which is defined by,

$$\begin{array}{c} {\hat{s}}_{i}\left(t\right)=s_{i}\left(t\right)+{𝒩}_{w}, \end{array}$$

where ${𝒩}_{w}$ is the additive white Gaussian noise (AWGN) with zero mean and standard deviation $\sigma_{w}$. Upon receiving ${\hat{s}}_{i}\left(t\right)$, the receiver performs the reverse process (i.e., demodulation, decoding, demapping, etc.) in order to construct the observed state. Let ${\hat{𝒪}}_{i}\left(t\right)$ be the reconstructed observed state which corresponds to ${𝒪}_{i}\left(t\right)$. The reconstructed observed state ${\hat{𝒪}}_{i}\left(t\right)$ is then fed to our proposed fusion algorithm (discussed in Section 4) to track the original state of the wind turbine. The overall communication framework is depicted in Figure 2. In the following, we briefly describe the repeat-accumulate code along with its encoding and decoding procedures.

### Repeat Accumulate (RA) Codes

**Figure 3:**
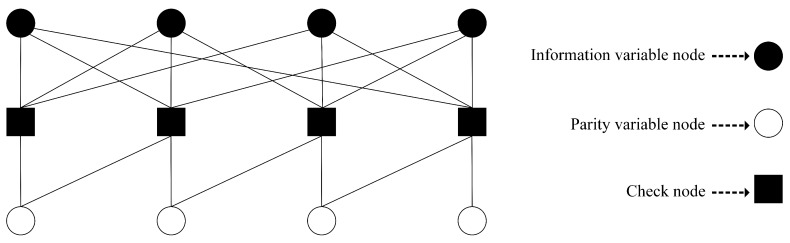
A Tanner graph representation of a repeat accumulate code with (q,a)=(3,3). In the graph, filled and unfilled circle nodes represent the information and parity bits, respectively, while rectangular nodes represent check equations.

Low-density parity-check (LDPC) codes are best known for their capacity approaching performance and low complexity decoding property [31,32]. Repeat accumulate (RA) codes are a special type of LDPC codes which inherit the above properties while allowing low encoding complexity [33,34,35]. Similar to the LDPC codes, RA codes can be represented by a bipartite graph with *K* information variable nodes, *M* parity variable nodes, and *M* check nodes. Each information node represents an information bit, while each check node represents a check equation satisfying the condition that the modulo 2 sum of all the connected nodes will be zero. The value of each parity node, which represents a redundant/parity bit, is generated such that the check equation satisfies the above condition. In an RA code, each information node is connected to more than one check node, while each parity bit node has connection with exactly two check nodes (except the last parity node, which connects to only one check node). We connect the parity bit nodes and the check nodes such that the $i^{th}$ parity bit node always connects with the check nodes at position $i^{th}$ and ${\left(i-1\right)}^{th}$. The parity/redundant bits or the value of the parity nodes are generated in the following manner. The $i^{th}$ parity bit node’s value is calculated by performing modulo-2 sum of the information bit nodes that have connection with the $i^{th}$ check node and the ${\left(i-1\right)}^{th}$ parity bit node. A RA code is referred to as (q,a)-regular code, when each of the information bit node is connected with exactly *q* check nodes and each of the check nodes is connected with exactly *a* information bit nodes. A Tanner graph representation of a (q,a)=(3,3) regular RA code is depicted in Figure 3. Note that a Tanner graph is equivalent to a binary matrix H of size M×(K+M), where each column and row represent variable and check nodes, respectively and each non zero entry represents a connection between the corresponding variable and check nodes.

#### Belief Propagation Decoding

A belief propagation (BP) decoding algorithms, which belong to the category of message passing algorithms, is among the best known decoding algorithms over binary input AWGN channels [36]. In a BP decoding algorithm, messages that passed between nodes are represented by log likelihood ratios (LLRs) (see Equations (3)–(5) in the following). Let $x_{m}$ be the $m^{th}$ bit of a codeword (i.e., $m^{th}$ variable node in the Tanner graph) and $y_{m}$ be the corresponding channel output. For variable node *m*, the initial (channel) LLR is given by ([37] [Chapter 2]),

(3) $$\begin{array}{c} r_{m}=\log\frac{P_{\mathrm{ch}}\left(y_{m}|x_{m}=0\right)}{P_{\mathrm{ch}}\left(y_{m}|x_{m}=1\right)}. \end{array}$$

For simplicity, we use the following notations to describe the BP decoding algorithm:

- $S_{v}\left(m\right)$ → set of variable nodes that have connection/edge with the $m^{th}$ check node.
- $S_{c}\left(m\right)$ → set of check nodes that have connection/edge with the $m^{th}$ variable node.
- $V_{n,m}^{\left(\ell \right)}$ → LLR message sent from variable node *m* to check node *n* at iteration *ℓ*.
- $C_{n,m}^{\left(\ell \right)}$ → LLR message sent from check node *n* to variable node *m* at iteration *ℓ*.

**Message from check node:**

(4) $$\begin{array}{c} C_{n,m}^{\left(\ell \right)}=2\tanh^{-1}\left(\prod_{m^{\prime }\in S_{v}\left(n\right),m^{\prime }\neq m}\tanh\left(\frac{V_{n,m^{\prime }}^{\left(\ell \right)}}{2}\right)\right). \end{array}$$

**Message from variable node:**

(5) $$\begin{array}{c} V_{n,m}^{\left(\ell +1\right)}=\sum_{n^{\prime }\in S_{c}\left(m\right),n^{\prime }\neq n}C_{n^{\prime },m}^{\left(\ell \right)}+r_{m}. \end{array}$$

The above decoding process is initialized by sending the channel LLRs from each variable node to the connected check nodes. After maximum iteration, the $\ell_{\max}$, $m^{th}$ decoded bit is given by

(6) $$\begin{array}{c} {\hat{x}}_{m}=\left\{\begin{array}{c} 0\;\;\mathrm{if}\;\sum_{n\in S_{c}\left(m\right)}C_{n,m}^{\left(\ell_{\max}\right)}+r_{m}\geq 0\\ 1\;\;\mathrm{if}\;\sum_{n\in S_{c}\left(m\right)}C_{n,m}^{\left(\ell_{\max}\right)}+r_{m}<0 \end{array}\right. \end{array}$$

A successful decoding event can be indicated by summed syndrome ψ, which is defined by $\psi =\sum {\mathrm{mod}}_{2}\left(H*{\hat{x}}^{T}\right)$, where ${\mathrm{mod}}_{2}\left(\cdot \right)$ is a modulo-2 operation and $\hat{x}={\hat{x}}_{1},{\hat{x}}_{2},\ldots ,{\hat{x}}_{K+M}$. ψ=0 indicates a successful decoding event, while ψ>0 indicates an unsuccessful decoding event.

## 4. Proposed Sensor Fusion Technique

We now present our state estimation with sensor fusion based on a Kalman filter algorithm [38,39]. We illustrate the proposed scheme in the following four steps:

**Step 1—Prediction:** Let ${\hat{X}}^{-}\left(t\right)$ and ${\hat{P}}^{-}\left(t\right)$ be the predicted state and co-variance matrix, respectively. According to the Kalman filter algorithm, we calculate ${\hat{X}}^{-}\left(t\right)$ and ${\hat{\Upsilon }}^{-}\left(t\right)$ by

$$\begin{array}{c} {\hat{X}}_{i}^{-}\left(t\right)=A_{d}{\hat{X}}_{i}\left(t-1\right)+B_{d}U\left(t-1\right)\\ \Upsilon_{i}^{-}\left(t\right)=A_{d}\Upsilon_{i}\left(t-1\right)A_{d}^{T}+Q \end{array}$$

We denote $\Upsilon_{i}\left(t\right)$ as the $i^{th}$ local estimator’s updated co-variance matrix at time step *t*. Initially we set $\Upsilon_{i}\left(0\right)$ to a predefined value $\Upsilon_{0}$.

**Step 2—Modification:** We find the summed syndrome value of each component of ${\hat{𝒪}}_{i}\left(t\right)$. We remove the erroneous component from ${\hat{𝒪}}_{i}\left(t\right)$ and modify $C_{i}$ and $R_{i}$ accordingly. Let ${\bar{𝒪}}_{i}\left(t\right)$, ${\bar{C}}_{i}\left(t\right)$, and ${\bar{R}}_{i}\left(t\right)$ be the modified version of ${\hat{𝒪}}_{i}\left(t\right)$, $C_{i}$ and $R_{i}$, respectively.

**Step 3—Update:** Let $\epsilon_{i}\left(t\right)$ and ${𝒢}_{i}\left(t\right)$ be the measurement pre-fit residual and Kalman gain, respectively. We calculate $\epsilon_{i}\left(t\right)$ and ${𝒢}_{i}\left(t\right)$ by

$$\begin{array}{c} \epsilon_{i}\left(t\right)={\bar{O}}_{i}\left(t\right)-{\bar{C}}_{i}\left(t\right){\hat{X}}_{i}^{-}\left(t\right)\\ {𝒢}_{i}\left(t\right)=\frac{\Upsilon_{i}^{-}\left(t\right){\bar{C}}_{i}{\left(t\right)}^{T}}{{\bar{C}}_{i}\left(t\right)\Upsilon_{i}^{-}\left(t\right){\bar{C}}_{i}{\left(t\right)}^{T}+Q} \end{array}$$

At time step *k*, co-variance matrix estimate and the updated state estimate are calculated by,

$$\begin{array}{c} \Upsilon \left(t\right)=\Upsilon^{-}\left(t\right)-𝒢\left(t\right){\bar{C}}_{i}\left(t\right)\Upsilon_{i}^{-}\left(t\right)\\ {\hat{X}}_{i}\left(t\right)={\hat{X}}_{i}^{-}\left(t\right)+{𝒢}_{i}\left(t\right){\bar{R}}_{i}\left(t\right) \end{array}$$

**Figure 4:**
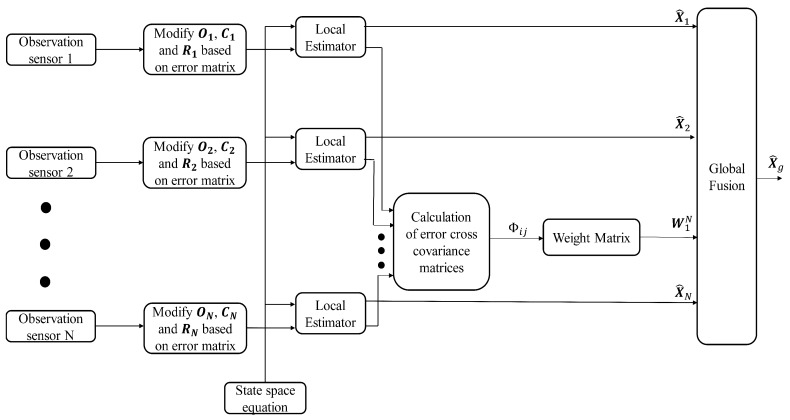
Block diagram of proposed sensor fusion technique.

**Step 4—Fusion:** Let $\Phi_{pq}\left(t\right)$, p,q∈{1,2,…,N} be the error cross co-variance between the $p^{th}$ and the $q^{th}$ sensors. $\Phi_{pq}\left(t\right)$ is given by

(7) $$\begin{array}{c} \Phi_{pq}\left(t|t\right)=\left\{\begin{array}{cc} \Upsilon_{0} & \;\mathrm{if}\;t=0\\ \Upsilon_{p}\left(t\right) & \;\mathrm{if}\;m=n\\ I_{\lambda }-{𝒢}_{p}\left(t\right){\bar{C}}_{p}\left(t\right)]\times & \mathrm{Otherwise}\\ A_{d}\Phi_{pq}\left(t-1|t-1\right)A_{d}^{T}+\Gamma Q\Gamma^{T}]\times \\ I_{\lambda }-{𝒢}_{q}\left(t\right){\bar{C}}_{q}\left(t\right)] \end{array}\right. \end{array}$$

where $I_{\lambda }$ is the identity matrix of size λ×λ and λ is the number of components in X. Following [18,40], optimal fusion based on the linear minimum variance can be written as

(8) $$\begin{array}{c} {\hat{X}}_{g}\left(t\right)=W_{1}\left(t\right){\hat{X}}_{1}\left(t\right)+W_{2}\left(t\right){\hat{X}}_{2}\left(t\right)+\ldots +W_{N}\left(t\right){\hat{X}}_{N}\left(t\right) \end{array}$$

where the matrix weight $\bar{W}\left(t\right)$ is given by

(9) $$\begin{array}{c} \bar{W}\left(t\right)=\Xi {\left(t\right)}^{-1}ℐ{\left({ℐ}^{T}\Xi \left(t\right)ℐ\right)}^{-1} \end{array}$$

where $\bar{W}\left(t\right)={\left[W_{1}\left(t\right),W_{2}\left(t\right),\ldots ,W_{N}\left(t\right)\right]}^{T}$ is an λN×λ matrix, $\Xi \left(t\right)=(\Phi_{pq}\left(t\right))$, p,q=1,2,…,N is an λN×λN symmetric positive definite matrix, and $ℐ={\left[I_{\lambda },\ldots ,I_{\lambda }\right]}^{T}$ is an λN×λ matrix. The overall fusion process is shown in Figure 4.

## 5. Performance Evaluations

In this section, we present the performance of the proposed communication and fusion technique. We also compare the numerical results with a traditional fusion technique [18]. We built a simulation environment in Matlab to evaluate the tracking performance of the wind turbine’s state. The parameters of the induction generator are shown in Table 2. We set the following values for the input parameters: the stator voltage in the *d* axis is $v_{ds}=0.04$ Volt, the stator voltage in the *q* axis is $v_{qs}=0.99$ Volt, the rotor voltage in the *d* axis is $v_{dr}=0.02$ Volt, and the rotor voltage in the *q* axis is $v_{qr}=0.206$ Volt. Note that the above parameters are adopted from the experimental setup reported in [28]. The process’ noise co-variance is set to $Q=0.95I_{\lambda }$ with constant matrix $\Gamma =I_{\lambda }$. For the following results, simulations were carried out for 150 steps with a step size of Δt=0.0001 s.

**Figure 5:**
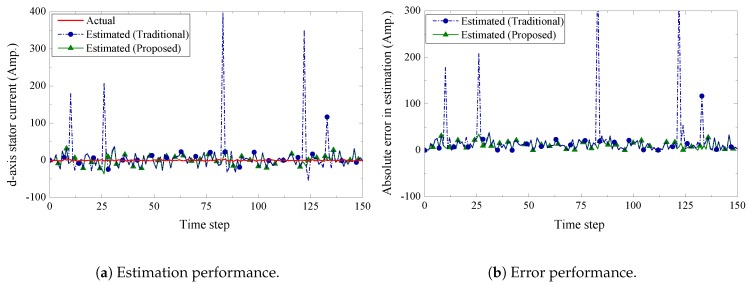
d-axis stator current estimation and error performance.

**Figure 6:**
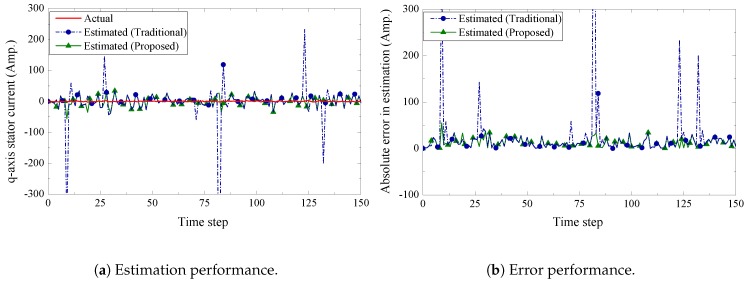
q-axis stator current estimation and error performance.

**Figure 7:**
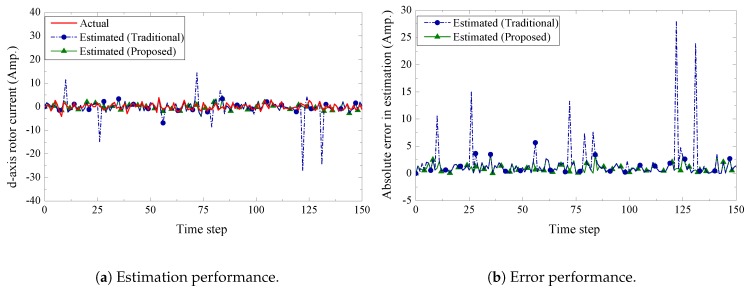
d-axis rotor current estimation and error performance.

**Figure 8:**
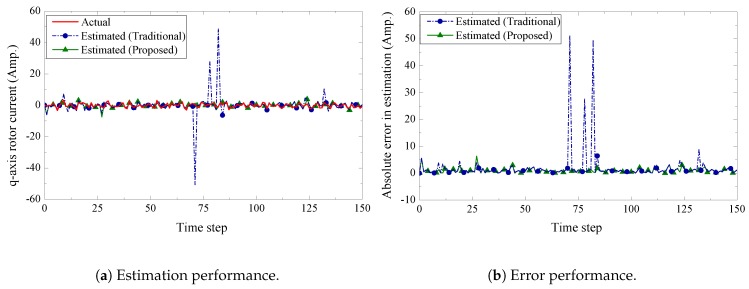
q-axis rotor current estimation and error performance.

**Figure 9:**
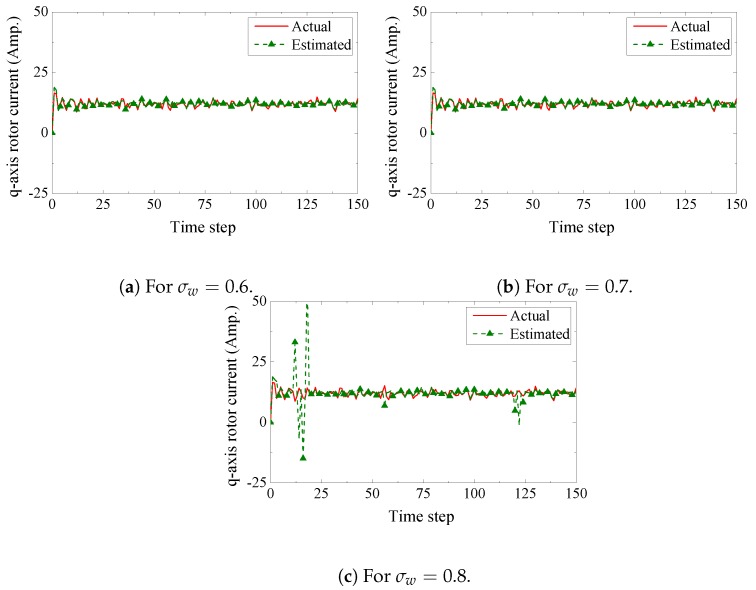
Impact of wireless channel quality on estimation performance.

We present the estimation performance of the proposed scheme in Figure 5, Figure 6, Figure 7 and Figure 8. Four sensors are used to measure the four current components of the induction generator with the following measurement matrices $C_{1}=\left[1\;0\;0\;0\right]$, $C_{2}=\left[0\;1\;0\;0\right]$, $C_{3}=\left[0\;0\;1\;0\right]$, $C_{4}=\left[0\;0\;0\;1\right]$. The measurement noise co-variances of the four sensors are set as 1.6, 1.2, 1.4, and 1.6, respectively. We consider a 32-bits uniform quantizer to map each component of the measured state. We use a rate $\frac{1}{2}$-(q,a)=(4,4) repeat-accumulate code over each mapped 32 bit frame and hence each component is represented by a codeword of 64 bits. This codeword is then modulated with BPSK and sent over a wireless channel with noise standard deviation 0.7. From the results, we observe that the proposed fusion technique can track the state of the wind turbine very closely. As a benchmark, we also present results obtained from a traditional fusion algorithm [18]. We show that our proposed scheme can significantly outperform the traditional estimation approach. In case of an error event due to the wireless transmission, it is expected that the measured state received by the remote receiver will be unreliable. While the traditional fusion algorithm treats all the measured states with the same importance, the proposed algorithm ignores the measured state and gives priority to the observation from the system dynamics. We notice that the traditional fusion algorithm fails to track the state (blue spikes in the figures), which are results of erroneous transmissions. We also investigate the impact of the wireless channel on the estimation performance of the proposed scheme. In Figure 9, we show the estimation performance while varying the channel noise standard deviation $\sigma_{w}$. Note that in terms of noise standard deviation, the decoding threshold of the presented (4,4)-RA code is 0.885. For $\sigma_{w}=0.6$ and $\sigma_{w}=0.7$, we observe similar estimation performance, which indicates that the wireless channel does not influence the estimation performance when the noise standard deviation is well below the threshold. However, when the noise standard deviation is close to the threshold (for $\sigma_{w}=0.8$), we observe inaccuracies in the estimation results.

## 6. Conclusions

The communication link between wind turbines and control center is often wireless to the remote placement of wind farms. In this paper, we have presented the state estimation of a wind turbine while taking into account the uncertainty of the wireless channel. We have shown an IoT inspired wireless communication framework to monitor the states of the wind turbine. A repeat-accumulate coded communication scheme is presented to tackle the noise induced by the unreliable wireless channel. Moreover, we have proposed an effective fusion algorithm to process and combine the multiple readings from different sensors of a wind turbine. Through simulation results, we have shown that our proposed scheme can track the state of a wind turbine accurately. We have also shown that the estimation performance of the proposed scheme outperforms estimation performance of traditional fusion algorithms. In the future we aim to apply our proposed scheme in a practical test-bed to evaluate the performance for real-world use cases.

## Figures and Tables

**Table 1:** Comparison of different works on state estimation of wind turbine.

Works	Type of Wind Turbine	Filter Type	Sensor Fusion	Impact of Wireless Channel	Error Correction Technique
Berg et al. [6]	Generic	Linear Kalman	No	No	No
Ritter et al. [8]	Generic	Linear Kalman	No	No	No
Petar et al. [9]	Generic	Extended Kalman	No	No	No
Bourlis et al. [10]	Generic	Adaptive Kalman	No	No	No
Blanco et al. [11]	Generic	Extended Kalman	No	No	No
Sudev et al. [12]	Generic	Particle filter	No	No	No
Yu et al. [13]	DFIG	Unscented Kalman	No	No	No
Yu et al. [14]	DFIG	Unscented Kalman	No	No	No
Prajapat et al. [15]	DFIG	Unscented Kalman	No	No	No
Shahriari et al. [16]	PMSG	Extended Kalman	No	No	No
This work	Generic	Linear Kalman	Yes	Yes	Yes

**Table 2:** Induction Generator Parameters.

Parameter	Value
Base frequency	10 Hz
Stator frequency	15 Hz
Rotor frequency	15 Hz
Resistance of stator	0.004 Ω
Resistance of rotor	0.005 Ω
Reactance of stator	0.09 Ω
Reactance of rotor	0.08 Ω
Magnetizing reactance	3.95 Ω
